# Evaluation of the Effect of Different Doses of Low Energy Shock Wave Therapy on the Erectile Function of Streptozotocin (STZ)-Induced Diabetic Rats

**DOI:** 10.3390/ijms140510661

**Published:** 2013-05-21

**Authors:** Jing Liu, Feng Zhou, Guang-Yong Li, Lin Wang, Hui-Xi Li, Guang-Yi Bai, Rui-Li Guan, Yong-De Xu, Ze-Zhu Gao, Wen-Jie Tian, Zhong-Cheng Xin

**Affiliations:** 1 Andrology Center, Peking University First Hospital, Peking University, Beijing 100034, China; E-Mails: liujing827@yahoo.cn (J.L.); linwang09@yahoo.cn (L.W.); huixilee@gmail.com (H.-X.L.); bgy73kr@hotmail.com (G.-Y.B.); guanruili@gmail.com (R.-L.G.); xyongde@gmail.com (Y.-D.X.); gaozhezhu@gmail.com (Z.-Z.G.); 2 Department of Urology, First Affiliated Hospital of Soochow University, Suzhou 215006, Jiangsu, China; E-Mail: zhoufeng0319@163.com; 3 Department of Urology, General Hospital of Ningxia Medical University, Yinchuan 750004, Ningxia, China; E-Mail: guangyongli1979@hotmail.com; 4 Department of Urology, China-Japan Union Hospital of Jilin University, Changchun 130033, Jilin, China; E-Mail: tianwenjie87@hotmail.com

**Keywords:** erectile dysfunction, diabetes mellitus, low energy shock wave therapy, corpus cavernosum

## Abstract

To investigate the therapeutic effect of different doses of low energy shock wave therapy (LESWT) on the erectile dysfunction (ED) in streptozotocin (STZ) induced diabetic rats. SD rats (*n* = 75) were randomly divided into 5 groups (normal control, diabetic control, 3 different dose LESWT treated diabetic groups). Diabetic rats were induced by intra-peritoneal injection of STZ (60 mg/kg) and rats with fasting blood glucose ≥ 300 mg/dL were selected as diabetic models. Twelve weeks later, different doses of LESWT (100, 200 and 300 shocks each time) treatment on penises were used to treat ED (7.33 MPa, 2 shocks/s) three times a week for two weeks. The erectile function was evaluated by intracavernous pressure (ICP) after 1 week washout period. Then the penises were harvested for histological study. The results showed LESWT could significantly improve the erectile function of diabetic rats, increase smooth muscle and endothelial contents, up-regulate the expression of α-SMA, vWF, nNOS and VEGF, and down- regulate the expression of RAGE in corpus cavernosum. The therapeutic effect might relate to treatment dose positively, and the maximal therapeutic effect was noted in the LESWT300 group. Consequently, 300 shocks each time might be the ideal LESWT dose for diabetic ED treatment.

## 1. Introduction

Erectile dysfunction (ED) is not only a health issue, which seriously impacts the quality of life (QOL) of men and their spouses or partners. Diabetes is a serious and growing public health problem that results in reduced QOL significantly. It is estimated that 366 million people had diabetes mellitus (DM) in 2011. By 2030 this would have risen to 552 million [1]. DM is a leading cause of ED, affecting ≥50% of men with DM worldwide [2]. The prevalence of ED in diabetics is three times higher than in men without DM [3,4].

PDE5 inhibitors (PDE5i) came into the market in 1998, which revolutionized ED treatment on demanded as the first-line treatment option [5]. However, PDE5i showed less efficacy in diabetic patients with ED [6] and there is no evidence to suggest that PDE5i could improve pathological changes of penises in patients with ED.

Others and our previous studies found that the pathological changes in diabetic rats include the corpus cavernosum fibro-muscular pathological changes, endothelial dysfunction and neuropathy [7,8].

Research on gene therapy and stem cell therapy on ED showed the potential effects on regulation corpus cavernosal pathological changes; however, these therapies still need a long way to go until clinical application is possible [9,10].

Extracorporeal shock wave therapy was applied in the medical field in the 1980s, and was first used for successfully treating urinary stones. After that, high energy shock wave therapy developed rapidly, and it was used for bill duct stones [11], pancreatic stones [12], stones of the parotid gland [13], stomach stones, nonunion [14], pseudarthrosis, avascular necrosis, muscle bone and joint pain diseases [15]. Untill 2000, Gutersohn discovered that low energy shock waves could up-regulate angiogenesis factors in cultured human epithelial cells [16], and later it was found that low energy shock waves could promote neovascularization to improve blood supply [17]. Thus, low energy shock wave therapy (LESWT) was applied in medical care more and more widely. And now, LESWT has been applied in the treatment of myocardial ischemial [18], Peyronie disease [19], diabetic foot, promoting incision healing [20], weight loss [21] and ED [22].

Although LESWT was reported to be effective, minimally invasive or non-invasive in the treatment of ED patients, it is not a fully-fledged, accredited treatment programs [23].

In order to investigate the ideal dose and the mechanism of LESWT on the treatment of ED, the therapeutic effect of different doses of LESWT on STZ induced diabetic ED rats was evaluated.

## 2. Results and Discussion

### 2.1. Effect of LESWT on Erectile Function

**Figure 1:**
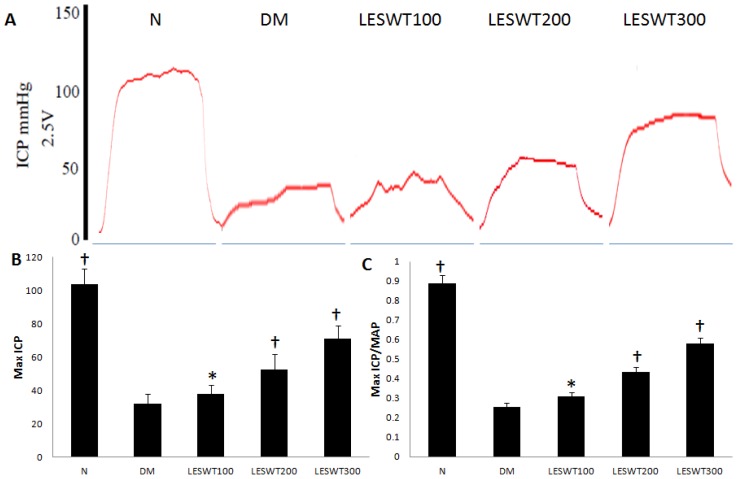
Erectile function assessed by Max intracavernous pressure (ICP) and ICP/mean arterial pressure (MAP). (**A**) Representative ICP responses to electrical stimulation of the cavernous nerve for age-matched normal control, diabetic and low energy shock wave therapy (LESWT) treated (100, 200, 300 shocks each time) diabetic groups. The stimulus interval (60 s) was indicated; (**B**) Max ICP in 5 groups; (**C**) Max ICP/MAP in 5 groups. * *p* < 0.05, ^†^*p* < 0.01 compared with the diabetes mellitus (DM) group.

The erectile function was assessed by Max ICP and ICP/MAP (intracavernous pressure/mean arterial pressure). The results showed that both Max ICP and ICP/MAP revealed a significant decrease in DM rats compared to normal control rats (*p* < 0.01). However, the Max ICP and ICP/MAP was significantly improved in different doses of LESWT treated DM rats compared to DM control rats. The maximal efficacy was noted in the LESWT300 group (Figure 1).

### 2.2. Effect of LESWT on Extracellular Matrix (ECM) in Corpus Cavernosum

**Figure 2:**
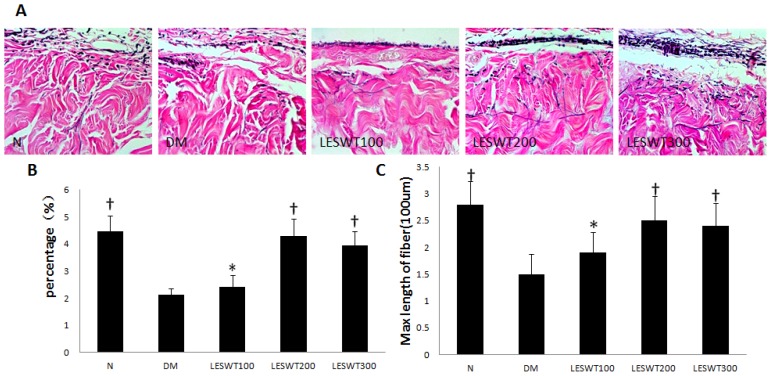
Elastic fiber in corpus cavernosum. (**A**) Representative photographs of cross-sections of corpus cavernosum with Hart’s elastic stain. Elastic fiber manifested as black, connective tissue was pink-red; (**B**) Maximal length of elastic fiber; (**C**) Percentage of elastic fiber. Each bar depicts the mean values (± standard deviation) from *N* = 5 per group. * *p* < 0.05, ^†^*p* < 0.01 compared with the DM group.

Hart’s elastic fiber staining showed a significant decrease in the Max length and percentage of elastic fiber in corpus cavernosum of diabetic rat compared to that of normal rats (*p* < 0.01). However, LESWT improved both of them significantly (*p* < 0.01) (Figure 2).

**Figure 3:**
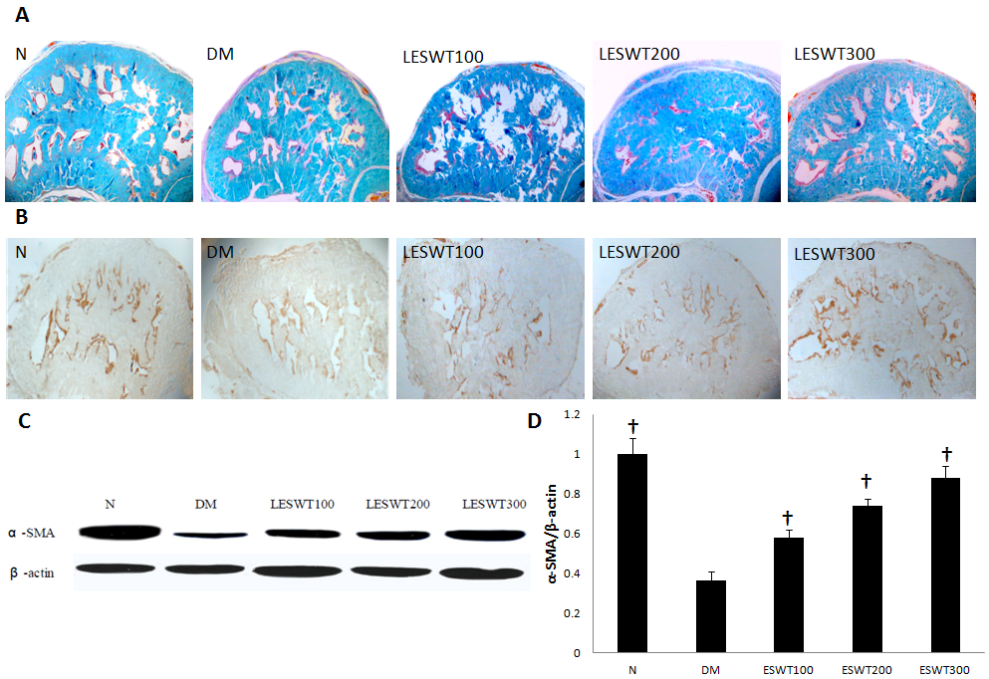
Smooth muscle contents in corpus cavernosum. (**A**) Masson trichrome staining. Smooth muscle manifested as red, connective tissue was green; (**B**) The expression of α-SMA by immunohistochemistry staining; (**C**) Western blot of α-SMA; (**D**) The ratio of α-SMA to β-actin by Western blot. Each bar depicts the mean values (± standard deviation) from *N* = 5 (immunohistochemical staining) and *N* = 3 (Western blots) animals per group. ^†^*p* < 0.01 compared with the DM group.

Masson trichrome staining was performed to assess extracellular matrix partly in corpus cavernosum. Corporal cavernosum tissue from diabetic rats showed lower smooth muscle/collagen ratio compared to normal rats, while LESWT could increase the smooth muscle/collagen ratio (Figure 3A).

### 2.3. Effect of LESWT on Smooth Muscle Contents in Corpus Cavernosum

We performed immunohistochemical staining, western blot with antibody to α-SMA and Masson trichrome staining to assess the smooth muscle contents. The results showed a significant reduction of smooth muscle contents in DM rats, *versus* normal control. However, LESWT significantly reversed it partly (*p* < 0.01). The therapeutic effect increased with the increase of the dose (Figure 3).

### 2.4. Effect of LESWT on Endothelium in Corpus Cavernosum

**Figure 4:**
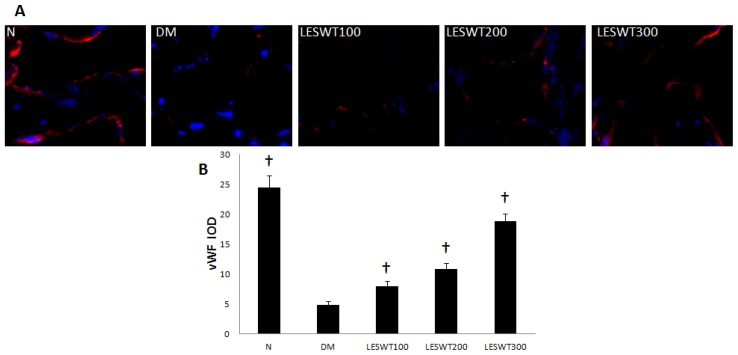
Endothelial contents in corpus cavernosum of low energy shock wave therapy (LESWT) treated diabetic rats. (**A**) The expression of von Willebrand Factor (vWF) by immunofluorescence staining; (**B**) Semiquantitative analysis of vWF expression. Each bar depicts the mean values (± standard deviation) from *N* = 5 animals per group. ^†^*p* < 0.01 compared with the DM group.

The immunofluorescence staining with antibody to vWF (Von Willebrand Factor) was performed to assess the endothelium. It showed that DM caused a significant reduction of endothelial contents in corpus cavernosum, but LESWT significantly reversed it (*p* < 0.01). The effect increased with the increase of treatment dose (Figure 4).

### 2.5. Effect of LESWT on the Expression of nNOS in Cavernous Nerve

**Figure 5:**
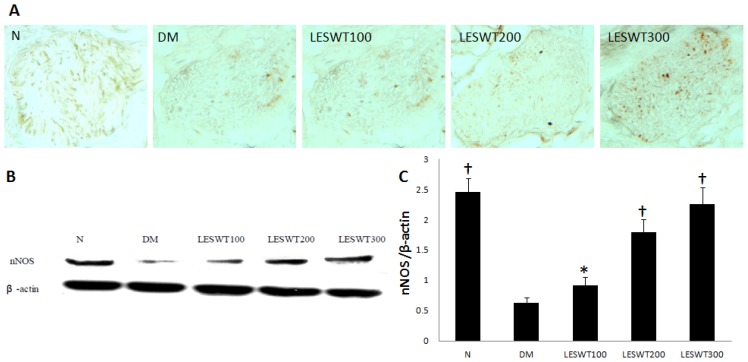
The expression of nNOS in penile dorsal nerve. (**A**) The expression of nNOS by immunohistochemistry staining; (**B**) Western blot of nNOS; (**C**) The ratio of nNOS to β-actin by Western blot. Each bar depicts the mean values (± standard deviation) from *N* = 5 (immunohistochemical staining) and *N* = 3 (Western blots) animals per group. * *p* < 0.05, ^†^*p* < 0.01 compared with the DM group.

The immunohistochemical staining and western blot with antibody to nNOS were performed to locate and quantify NOS-positive nerve fibers. The expression of nNOS in the penile dorsal nerves in DM was significantly less than in the normal control (*p* < 0.01). However, LESWT significantly improved the expression of nNOS (*p* < 0.01). The therapeutic effect increased with the increase of the dose (Figure 5).

### 2.6. Effect of LESWT on the Expression of VEGF in Corpus Cavernosum

**Figure 6:**
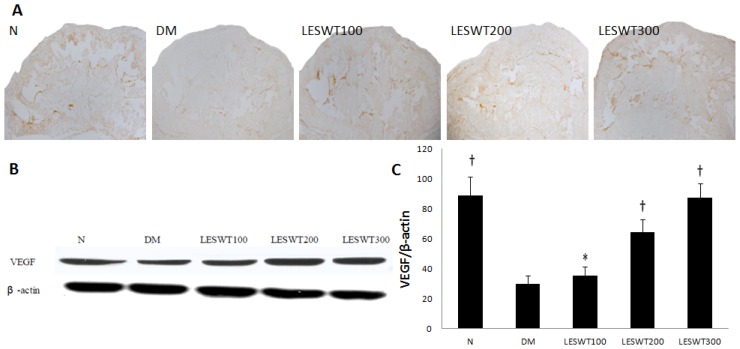
The expression of vascular endothelial growth factor (VEGF). (**A**) The expression of VEGF by immunohistochemistry staining; (**B**) Western blot of VEGF; (**C**) The ratio of VEGF to β-actin by Western blot. Each bar depicts the mean values (± standard deviation) from *N* = 5 (immunohistochemical staining) and *N* = 3 (Western blots) animals per group. * *p* < 0.05, ^†^*p* < 0.01 compared with the DM group.

The immunohistochemical staining and western blot with antibody to VEGF were performed to evaluate the expression of VEGF in penises. It showed a significant reduction of VEGF expression in corpus cavernosum of DM rats (*p* < 0.01). However LESWT significantly improved the expression of VEGF (*p* < 0.01). The effect was positively related to the treatment dose (Figure 6).

### 2.7. Effect of LESWT on the Expression of RAGE

**Figure 7:**
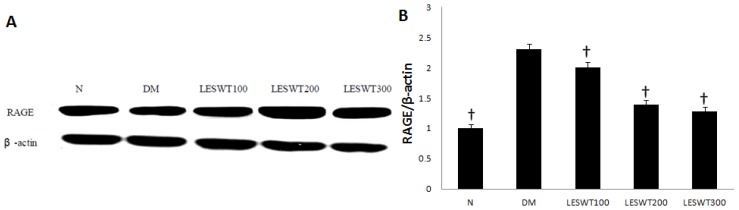
The expression of receptor for advanced glycation end products (RAGE). (**A**) Western blot of RAGE; (**B**) The ratio of RAGE to β-actin by Western blot. Each bar depicts the mean values (± standard deviation) from *N* = 3 (Western blots) animals per group. ^†^*p* < 0.01 compared with the diabetic control group.

The western blot with antibody to RAGE was performed to assess severity of DM. It showed that the expression of RAGE was significantly up-regulated in the DM group. However, LESWT treatment significantly decreased it (*p* < 0.01). The effect was positively related to the treatment dose (Figure 7).

ED is a common complication of diabetes. Clinical practice has no treatment modality specifically designed for the difficulty of treating diabetic ED due to the multifactorial and complex pathophysiology of development. LESWT was reported to be effective in improving local blood supplement [18]. Some clinical and experimental research proved that LESWT was effective in ED treatment [19,24]. The current study aims to investigate the effect and ideal dose of LESWT on the treatment of diabetic ED, and to elucidate its possible mechanism.

STZ was used to induce Type 1 DM rat model [25]. Twelve weeks after STZ injection, these diabetic rats had a poor erectile function in our study, and the mean Max ICP decreased to 32.3 mmHg, while that is about 103.8 mmHg in the normal control. However, LESWT improved the erectile function of diabetic rats in our study, and most of diabetic rats showed a therapeutic effect in response to LESWT treatment. The mean Max ICP of diabetic rats treated by different doses of LESWT increased to 38.1 mmHg in the LESWT100 group, 52.5 mmHg in the LESWT200 group and 70.9 mmHg in the LESWT300 group. These results indicated that the therapeutic effect might be positively correlated with the treatment dose of LESWT and the maximal efficacy of LESWT on erectile function was noted in the LESWT300 group.

Smooth muscle cells, endothelium and nerves in corpus cavernosum play an important role in penile erection [26]. Our previous study found that fibrosis of corpus cavernosum was an important pathological process in diabetic ED model, which showed a decrease of smooth muscle and endothelium, damage of nerves and increase of collagen [27]. In our study, α-SMA, vWF and nNOS were respectively used to assess smooth muscle, endothelium and nerves [28–30]. All of our data showed serious pathological reduction of smooth muscle, endothelium and nerves in coupus cavernous of diabetic rats. However, LESWT treatment could significantly restore these pathological changes. In addition, collagen and elastic fibers of the tunica albuginea are key components of this compliant tissue and permit an increase in girth and length during tumescence and rigidity of the penis. The changes of length and contents of elastic fiber may have an impact on the increase of the girth and length of the penis when erection.

VEGF is a key regulator of physiological angiogenesis [31]. It has been proved that LESWT could up-regulate the expression of VEGF and induce neovascularization without any adverse effects in treatment of human myocardial ischemia [18,32]. In this study, LESWT treatment significantly improved smooth muscle and endothelium contents, and the expression of VEGF. So, we speculated that up-regulation of VEGF might improve blood supply in the penis and this would be helpful for erection by restoring pathological changes. Recent research reported that the beneficial effects of LESWT in treatment of ED might be mediated by recruitment of endogenous MSCs(Mesenchymal stem cells) [33]. And the activation and/or recruitment of MSCs could up-regulate the expression of VEGF and restore pathological changes of smooth muscle and endothelium to improve the erectile function of diabetic rats. It was difficult to determine whether the expression of VEGF was up-regulated by LESWT directly or by activating MSCs. So, we should be more concerned about MSCs’ role in LESWT treatment.

Interestingly, the expression of RAGE in diabetic rats significantly increased. However, LESWT treatment could decrease the expression of RAGE in the penis. RAGE, the receptor for advanced glycation end product (AGE), plays a vital role in the development of DM. The binding of AGEs and RAGE activates the nuclear factor-κB (NF-κB) and other signaling pathways to cause pathological changes in diabetic complication [34]. The mechanism of LESWT on down-regulation of RAGE remains unclear. However, what we could be certain is that the reduction of RAGE would be beneficial for improving the microenviroment in the penis.

In summary, different doses of LESWT treatment could improve erectile function by restoring fibro-muscular pathological changes of smooth muscle, endothelium and nerves in corpus cavernosum of diabetic rats. Furthermore, LESWT could up-regulate the expression of VEGF and down-regulate the expression of RAGE, which might improve microenviroment and be helpful to the erectile function. The maximal therapeutic effect was noted in the LESWT300 group. Consequently, 300 shocks each time might be the ideal LESWT dose for diabetic ED treatment.

## 3. Experimental Section

### 3.1. Animals

A total of 75 SD rats (male, 8 week, 250–300 g) were randomly distributed into 5 groups (normal control, diabetes control, LESWT100, LESWT200 and LESWT300) in this study. The experiment performance was approved by the Institutional Animal Care and Use Subcommittee of Peking University. Fasted for 16 h, 60 SD rats were injected intraperitoneally with freshly prepared streptozotocin (Sigma Chemical Co., St. Louis, MO, USA) (60 mg/kg) while 15 rats in normal control were injected with saline. Blood glucose was detected 72 h after injection, at a regular interval of every week throughout the study. Blood glucose was measured using a blood glucose meter (B. Braun, Melsungen AG, Germany). Only rats with fasting glucose concentrations ≥300 mg/dL were included in the diabetic groups.

### 3.2. LESWT Treatment

Twelve weeks after induction of diabetes, rats in LESWT groups were anesthetized, in a supine position, lower abdomen shaved and penises were drawn out of the prepuce. Apply ultrasonic gel on the penis, then shock wave applicator (HaiBing medicial equipment limited corporation, Zhanjiang Economic and Technological Development Zone, Guangdong, China) was placed on the penises and a total of 100, 200 and 300 shocks were delivered according their groups (7.33 MPa, 2 shocks/s). Each groups received LESWT treatment three times a week for two weeks with a one- week washout period.

### 3.3. Mesurement of Erectile Function

We evaluated the erectile function of 6 rats each groups by cavernous nerve electrical stimulation after LESWT treatment. The major pelvic ganglions and cavernous nerves were exposed under anesthesia. After the skin overlying the penis was cut, two 23-gauge needles connected to PE-50 tube with heparinized saline (250 IU/mL) were inserted into the crus and left carotid artery. The other end of the PE-50 tube was connected to Biopac Systems MP150. The cavernous nerve was exposed and electrostimulation (12 Hz, pulse width 5 ms, 2.5 V, duration 60 s) of the cavernous nerve was applied with a stainless steel bipolar hook electrode. Mean arterial pressure (MAP) was measured concurrently. The ratio of Max ICP to MAP was calculated to normalize for variations in systemic blood pressure between subject rats.

### 3.4. Histochemistry and Immunohistochemistry

The penises were harvested and immersed in neutral buffered formalin containing 4% formaldehyde for 6 h, embedded in paraffin in vertical direction and cut in cross-section. Sections of 6 μm thickness were cut using a rotor microtome and were prepared for histochemistry and immunohistochemistry.

For Masson trichrome staining, sections were deparaffinized and hydrated by sequential incubations in xylene and ethanol. After washing, sections were then placed in Bouin fixative for 1 h at room temperature, transferred to 4% ferric ammonium sulfate for 5 min at 50 °C, and rapidly rinsed with distilled water at 50 °C. Slides were immersed in 0.1% acid fuchsin for 1 min and gently rinsed by repeatedly immersing in water 5 times, then placed in 1% phosphomolybdic acid for 10 min and then stained for 90 s in 0.25% light green/0.5% phosphomolybdic acid. The slides were washed in water until the rinses became clear and then dehydrated in graded ethanol, cleared with xylene and coverslipped using neutral resin. Images of tissue sections were captured with a digital camera and imported into Image Pro Plus 6.0 (Media Cybernetics, Inc., Bethesda, MD, USA).

For Hart’s elastic staining (*N* = 5 per group), penile tissue sections were soaked in 0.25% potassium permanganate solution for 5 min, cleared in 5% oxalic acid and soaked in resorcin–fuchsin solution (Poly Scientific, Bay Shore, NY, USA) overnight. After washing, sections were counterstained with tartrazine. Hart’s-stained sections were captured with a digital camera and imported into Image Pro Plus software.

For immunohistochemistry (*N* = 5 per group), Penile tissue sections were deparaffinized and hydrated. After washing in PBS for 5 min 3 times, the sections were blocked with 3% H_2_O_2_ for 10 min to quench endogenous peroxidase activity and with heat-induced epitope retrieval methods to perform the antigen unmasking. The tissue sections were incubated with antibody to alpha smooth muscle actin (α-SMA, Rabbit polyclonal, Abcam, Cambridge, MA, USA; 1:800), nNOS (Rabbit polyclonal, Santa Cruz Biotechnology, Inc., San Cruz, CA, USA; 1:1000) and vascular endothelial growth factor (VEGF, Rabbit polyclonal, Abcam, Cambridge, MA, USA; 1:400). The cell nucleus was performed with hematoxylin. Semiquantitative analysis was performed to evaluate the integrated optical dose (IOD) of α-SMA, nNOS and VEGF staining by the use of Image-Proplus software.

For immunofluorescence (*N* = 5 per group), the tissues were cryoprotected in sucrose, frozen and sectioned at 8 μm in a cryostat. Slides were incubated successively with blocking solution. The tissue sections were incubated with primary antibody to Rabbit polyclonal to Von Willebrand Factor (vWF, Abcam, Cambridge, MA, USA; 1:400). After the hybridization of secondary antibodies, and DAPI staining for the cell nucleus, the sections were observed at the fluorescence microscope (Leica DM 6000 Laser Station). Semiquantitative analysis was performed to evaluate the dose of vWF staining by the use of Image pro plus software 6.0 (Media Cybernetics, Silver Spring, MD, USA).

### 3.5. Western Blot

Penile tissue protein samples were prepared by homogenizing in RIPA lysis buffer. Equal amounts of protein (20 μg/mL) were electrophoresed on 10% SDS-PAGE and then transferred to polyvinylidene fluoride membrane (MilliporeCorp, Bedford, MA, USA). The membrane was blocked with 5% skimmed milk for 1 h at room temperature and incubated overnight at 4 °C with primary antibody to β-actin (Mouse polyclonal, Santa Cruz Biotechnology, CA, USA. 1:4000), α-SMA (Rabbit polyclonal, Abcam, Cambridge, MA, USA; 1:1000), Vascular endothelial growth factor (VEGF, Rabbit polyclonal, Abcam, Cambridge, MA, USA; 1:1000), nNOS (Rabbit polyclonal, Santa Cruz Biotechnology, CA, USA; 1:200) and receptor for advanced glycation end products (RAGE, Rabbit polyclonal, Abcam, Cambridge, MA, USA; 1:1000). After the hybridization of secondary antibodies, the resulting images were analyzed with ChemiImager 4000 (Alpha Innotech Corporation, San Leandro, CA, USA) to determine the integrated density value of each protein band. Results were semi-quantified by densitometry (*N* = 3 per group).

### 3.6. Statistical Analysis

Results are expressed as means ± standard deviation. One-way ANOVA followed by Bonferroni multiple comparisons’ test was used to evaluate whether differences between groups were significant. All calculations were performed using SPSS statistical software (version 13.0, SPSS, Chicago, IL, USA). Probability values of less than 0.05 were considered significant.

## 4. Conclusions

These results suggested that different doses of LESWT treatment could improve erectile function by restoring fibro-muscular pathological changes in corpus cavernosum of diabetic rats, include an increase of smooth muscle and endothelial contents and the expression of nNOS in nerves. And the best effect was noted in the LESWT300 group. So, 300 shocks each time might be the ideal LESWT dose for diabetic ED treatment.
